# Detection of "*Rickettsia *sp. strain Uilenbergi" and "*Rickettsia *sp. strain Davousti" in *Amblyomma tholloni *ticks from elephants in Africa

**DOI:** 10.1186/1471-2180-7-74

**Published:** 2007-08-07

**Authors:** Kotaro Matsumoto, Philippe Parola, Jean-Marc Rolain, Kathryn Jeffery, Didier Raoult

**Affiliations:** 1 Unité des Rickettsies, CNRS UMR 6020 IFR 48, Faculté de Médecine, Marseille, France; 2 Wildlife Conservation Society, Libreville, Gabon, Africa

## Abstract

**Background:**

To date, 6 tick-borne rickettsiae pathogenic for humans are known to occur in Africa and 4 of them were first identified in ticks before being recognized as human pathogens.

**Results:**

We examined 33 and 5 *Amblyomma tholloni *ticks from African elephants in the Central African Republic and Gabon, respectively, by PCR amplification and sequencing of a part of *gltA *and *ompA *genes of the genus *Rickettsia*. The partial sequences of *gltA *and *ompA *genes detected in tick in Gabon had 99.1% similarity with those of *R. heilongjiangensis *and 97.1% with those of *Rickettsia *sp. HL-93 strain, respectively. The partial *gltA *and *ompA *gene sequences detected in tick in the Central African Republic were 98.9% and 95.1% similar to those of *Rickettsia *sp. DnS14 strain and *R. massiliae*, respectively. Phylogenetic analysis showed *Rickettsia *sp. detected in Gabon clusters with *R. japonica *and *R. heilongjiangensis *in a phylogenetic tree based on the partial *gltA *and *ompA *genes. The genotype of the *Rickettsia *sp. detected in the Central African Republic is close to those of *R. massiliae *group in the phylogenetic tree based on partial *gltA *gene sequences, and distantly related to other rickettsiae in the tree based on partial *ompA *gene.

**Conclusion:**

The degrees of similarity of partial *gltA *and *ompA *genes with recognized species indicate the rickettsiae detected in this study may be new species although we could only study the partial sequences of 2 genes regarding the amount of DNA that was available. We propose the *Rickettsia *sp. detected in Gabon be provisionally named "*Rickettsia sp*. stain Davousti" and *Rickettsia *sp. detected in the Central African Republic be named "*Rickettsia sp. *strain Uilenbergi".

## Background

Spotted fever group (SFG) rickettsiae are obligatory intracellular Gram-negative bacteria that belong to the genus *Rickettsia*. They are associated with arthropods, mainly ticks, which act as vectors and/or reservoirs [1]. Between 1984 and 2005, 11 new species or subspecies of rickettsiae have been identified as emerging agents of human tick-borne diseases [1]. Additionally, many SFG rickettsiae have been identified only in ticks and their pathogenicity for humans is unknown [1].

To date, 6 tick-borne rickettsiae pathogenic for humans are known to occur in Africa [1]. *Rickettsia conorii conorii*, the agent of Mediterranean spotted fever, transmitted by *Rhipicephalus sanguineus *[2] is endemic in North Africa and has been reported from South Africa, Kenya and Zimbabwe. *R. conorii caspia*, the agent of Astrakhan fever, has recently been detected in *Rh. sanguineus *in Chad. It is now almost 15 years since *Rickettsia africae*, the agent of African tick-bite fever (ATBF), was discovered in sub-Saharan Africa. In southern Africa, *Amblyomma hebraeum*, a tick of large ruminants and wildlife species, is the recognized vector (and reservoir) of *R. africae*, which has also been detected in *A. variegatum *throughout west, central, and eastern sub-Saharan Africa, and in *A. lepidum *from the Sudan. *Rickettsia aeschlimannii *has been found in *Hyalomma marginatum marginatum *and *H. marginatum rufipes *and, while probably following the distribution of these ticks. This rickettsia has been identified in Morocco, Niger, Mali, Zimbabwe and South Africa. *Rickettsia massiliae *which was proven to be a human pathogen in 2005 has been detected in several *Rhipicephalus *ticks parasitizing cattle in the Central African Republic and Mali [1]. It should be noted that the four last rickettsiae were first identified in ticks before being recognized as human pathogens [1]. Finally, *R. rhipicephali*, a rickettsia of unknown pathogenicity, has been detected in ticks of the *Rh. compositus *group and *Rh. lunulatus *[3]. Here, we report two candidate new species in the genus *Rickettsia *detected in ticks collected from African elephants.

## Results

### Ticks

Thirty-eight ticks were collected from African elephants in the Central African Republic (5; 1 female, 3 males and 1 nymph) and Gabon (15 females and 18 males). They were all identified as *Amblyomma tholloni*.

### PCR and sequencing of *gltA *and *ompA *genes

One female from the Central African Republic and 1 male from Gabon were PCR positive for the rickettsial *gltA *and *ompA *genes. These amplicons were sequenced and ambiguous parts in the end of each sequence were deleted. Consequently, 914 bp of *gltA *and 590 bp of *ompA *genes, and 1196 bp of *gltA *and 544 bp of *ompA *genes were obtained from tick samples from Gabon and the Central African Republic, respectively. The partial sequences of the *gltA *and *ompA *genes of Gabon tick sample were 99.1% identical to those of *R. heilongjiangensis *[GenBank accession number, AY280709] and 97.1% to those of *Rickettsia *sp. HL-93 strain [AF179364], respectively. The partial sequences of the *gltA *and *ompA *genes amplified from the tick from the Central African Republic were 98.9% identical to those of *Rickettsia *sp. DnS14 strain [AF120028] and 95.1% identical to those of *R. massiliae *[U43799], respectively.

### Phylogenetic tree

**Figure 1:**
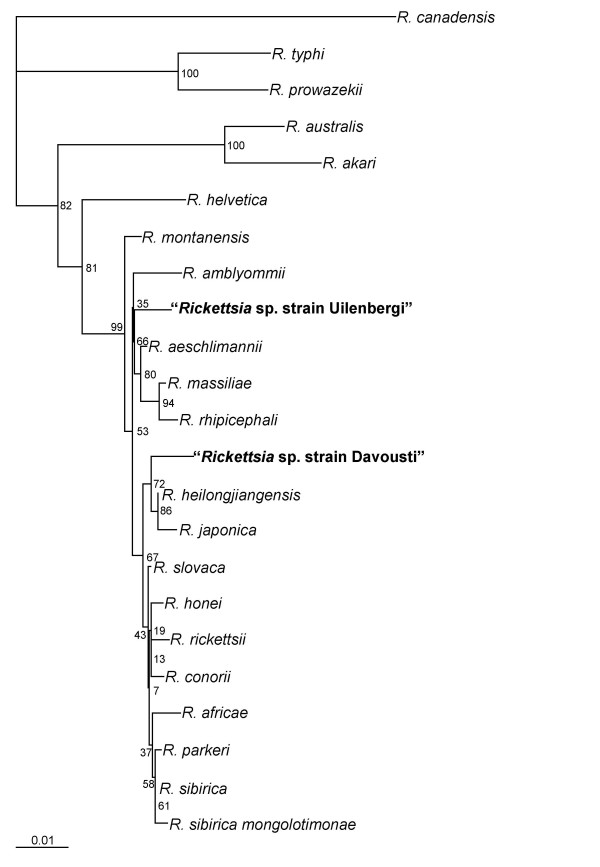
Phylogenetic tree based on *gltA *gene partial sequences. The GenBank accession numbers used to construct the phylogenetic tree were as follows: *R. parkeri*, U59732; *R. sibirica*, U59734; *R. sibirica mongolotimonae*, U59731; *R. africae*, U59733; *R. conorii*, U59730; *R. rickettsii*, U59729; *R. honei*, U59726; *R. slovaca*, U59725; *R. japonica*, U59724; *R. montanensis*, U74756; *R. massiliae*, U59719; *R. rhipicephali*, U59721; *R. aeschlimannii*, U59722; *R. helvetica*, U59723; *R. australis*, U59718; *R. akari*, U59717; *R. typhi*, U59714; *R. canadensis*, U59713; *R. prowazekii*, U59715; *R. amblyommii*, AY375163; *R. heilongjiangensis*, AY280709.

**Figure 2:**
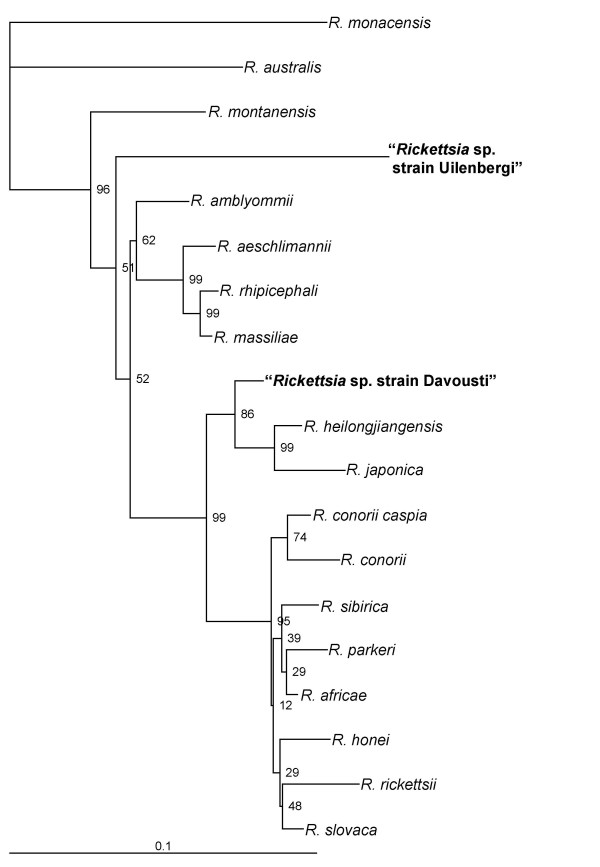
Phylogenetic tree based on *ompA *gene partial sequences. The GenBank accession numbers used to construct the phylogenetic tree were as follows: *R. australis*, AF149108; *R. heilongjiangensis*, AF179362; *R. honei*, U43809; *R. slovaca*, U43808; *R. sibirica*, U43807; *R. rickettsii*, U43804; *R. rhipicephali*, U43803; *R. parkeri*, U43802; *R. montanensis*, U43801; *R. aeschlimannii*, U43800; *R. massiliae*, U43799; *R. japonica*, U43795; *R. conorii*, U43806; *R. africae*, U43790; *R. amblyommii*, AY062007; *R. monacensis*, AF201329; and *R. conorii caspia*, AY112668.

Phylogenetic trees based on the partial sequences of *gltA *and *ompA *genes are shown in Figures 1 and 2, respectively.

## Discussion

To the best of our knowledge, this work provides the first molecular detection of spotted fever group rickettsiae in *A. tholloni*. This three-host tick is primarily found on African elephants [4,5] but also occurs on other animals including reptiles (lizards), birds and mammals (horse, antelope, wild pig, mongoose, buffalo and hippopotamus). It has been reported to feed on humans [4,5] and occurs widely in Africa following the distribution of the African elephant; from South Africa in the south to the Sudan in the north, and from Sierra Leone in the west to Somalia in the east [4,5]. *Amblyomma tholloni *can transmit *Ehrlichia ruminantium*, the agent of heartwater in domestic ruminants, but the tick has not been implicated in field outbreaks of the disease [6].

The rickettsiae we detected in *A. tholloni *in our study are members of the spotted fever group of rickettsiae. All the gene sequences that we have identified were unique. The rickettsia from Gabon clustered with *R. japonica *and *R. heilongjiangensis *in the phylogenetic trees based on the partial *gltA *and *ompA *genes (Figure 1 and 2). The rickettsia from the Central African Republic was close to the *R. massiliae *group in the phylogenetic tree based on the partial *gltA *gene sequences (Figure 1) and distinct from other rickettsiae in the tree based on the partial *ompA *gene (Figure 2). Recently, guidelines for the genotypic classification of rickettsiae at the genus, group, and species levels have been published [7]. According to these criteria, the degrees of nucleotide similarity with recognized species indicate the rickettsiae from the *A. tholloni *may be new species. However, we could only study the partial sequences of 2 genes regarding the amount of DNA that was available. Until isolates are made and definitively characterized as new species, we propose the rickettsia in the *A. tholloni *from Gabon be provisionally named "*Rickettsia *sp. strain Davousti" after the French veterinary scientist Bernard Davoust in recognition of his work on zoonoses. Further, we propose the rickettsia in the *A. tholloni *from the Central African Republic be provisionally named "*Rickettsia *sp. strain Uilenbergi" after the Dutch veterinary scientist Gerrit Uilenberg in recognition of his research into tick-borne tropical diseases. Both scientists coordinated field works that help to get the ticks used in our study.

The ticks harboring "*Rickettsia *sp. strain Davousti" and "*Rickettsia *sp. strain Uilenbergi" were feeding on elephants when they have been collected. It is not clear if the ticks were infected with the rickettsiae or if the organisms were only present in the ingested elephant blood. It appears unlikely the elephants were rickettsemic, however, as other ticks feeding on the same animals did not contain rickettsiae. It seems most probable that the organisms are associated with the ticks as it is commonly the case with other spotted fever group rickettsiae [8]. The tick vectors of the spotted fever group rickettsiae transmit the organisms while feeding and the role that the African elephant, the major host of *A. tholloni*, plays in the biology of the new rickettsiae requires further investigation. The pathogenicity of "*Rickettsia *sp. strain Davousti" and "*Rickettsia *sp. strain Uilenbergi" in the other species on which *A. tholloni *feeds, should also be investigate. In particular, *A. tholloni *is known to feed on people [4,5] and it would seem prudent to consider "*Rickettsia *sp. strain Davousti" and "*Rickettsia *sp. strain Uilenbergi" as potential human pathogens until further studies clarify the situation.

## Conclusion

The rickettsiae detected in *A. tholloni *in our study are members of the SFG rickettsiae, and their *gltA *and *ompA *gene sequences were unique. The degrees of similarity of partial *gltA *and *ompA *genes with recognized species indicate the rickettsiae detected in this study are new species. Although the pathogenicity of these rickettsiae were not evaluated in our study, it would seem prudent to consider these rickettsiae as potential human pathogens until further studies clarify the situation because *A. tholloni *is known to feed on people.

## Methods

### Ticks

Ticks were collected from African elephants (*Loxodonta africana*) in the Central African Republic in 1970 and in Gabon in 2004. In Gabon, ticks were collected from an elephant which had just been killed illegally by hunters. The ticks were identified on their morphology [4,5] and kept in 70% ethanol until DNA was extracted in Marseille, France, in 2005.

### DNA extraction and PCR for rickettsiae

The ticks were washed twice in distilled water and dried on sterile filter paper. Each tick was bisected longitudinally with a sterile scalpel blade and DNA extracted from one half using the QIAamp DNA Mini Kit (QIAGEN, Hilden, Germany) according to the manufacturer's instructions. PCR was performed using primers Rp877p and Rp1258n that amplify a 380 bp fragment of the *gltA *gene of rickettsiae [9]. DNA samples that gave a positive result with *gltA *PCR were also tested in a second PCR using primer pairs 190.70 or 190.180, and 190.701 that amplify a 632 bp and 552 bp fragment of the *ompA *gene of rickettsiae [10-12]. Negative controls consisted of (i) DNA from uninfected *Rh. sanguineus *(from our laboratory colony) which was extracted simultaneously with the test samples, and (ii) distilled water added to the PCR mix instead of DNA. One sample of DNA from *Rickettsia montanensis *was included in each PCR as a positive control

### Sequencing and extension of *gltA *sequences

Products amplified using PCR for rickettsial *gltA *and *ompA *were sequenced as described previously [9,12], used in a BLAST search [15]  and aligned with sequences of other rickettsial species registered in GenBank using Clustal program [13]. Sequences of partial *gltA *gene were extended using primers CS2d and CS535R, and CS409d and CS1273r as described previously [9,14]. Phylogenetic trees were constructed using TreeView [16] based on the data from ClustalW.

### Nucleotide sequence accession numbers

The partial *gltA *and *ompA *sequences of rickettsia in tick from Gabon have been deposited under accession number DQ402516 and DQ402517, respectively. The partial *gltA *and *ompA *sequences of rickettsia in tick from Central African Republic have been deposited under accession number DQ402514 and DQ402515, respectively.

## Authors' contributions

KM participated in PCR amplification and sequencing. PP identified tick species and coordinated the study. JMR participated in the analysis of sequences. KJ collected ticks in Gabon and the Central African Republic. DR designed the study. KM, PP, JMR and DR drafted the manuscript. All authors read and approved the final manuscript.
